# Perceived unfairness and socioeconomic inequalities in functional decline: the Dutch SMILE prospective cohort study

**DOI:** 10.1186/1471-2458-12-818

**Published:** 2012-09-22

**Authors:** Hans Bosma, Anouk Gerritsma, Gonnie Klabbers, Marjan van den Akker

**Affiliations:** 1 School for Public Health and Primary Care (CAPHRI), Department of Social Medicine, Maastricht University, P.O. Box 616, Maastricht, MD, 6200, the Netherlands; 2 School for Public Health and Primary Care (CAPHRI), Department of General Practice, Maastricht University, Maastricht, The Netherlands; 3 Department of General Practice, KU Leuven, Belgium

**Keywords:** Perceived unfairness, Socioeconomic differences in health, Health-related functioning

## Abstract

**Background:**

People in lower socioeconomic positions report worse health-related functioning. Only few examined whether perceptions of unfairness are particularly common in these people and whether this perceived unfairness relates to their subsequent poor health outcomes. We thus set out to examine the contribution of perceived unfairness to the higher risks of physical and mental dysfunction in men and women with a lower socioeconomic position.

**Methods:**

Seven-year prospective cohort data from the Dutch SMILE study among 1,282 persons, 55 years old and older, were used. Physical and mental health-related functioning was measured with the SF-36, socioeconomic status with income and education, and the perception of unfairness with an extended new measure asking for such perceptions in both work and non-work domains.

**Results:**

Perceived unfairness was more common in lower socioeconomic positions. Such perpection was related to both physical (odds ratio = 1.57 (95% confidence interval: 1.17-2.11)) and mental (1.47 (1.07-2.03)) decline, while low socioeconomic position was only related to mental decline (1.33 (1.06-1.67)). When socioeconomic position and perceived unfairness were simultaneously controlled, odds ratios for both determinants decreased only very little. Socioeconomic position and perceived unfairness were for the largest part independently related to longitudinal health-related decline.

**Conclusions:**

The general perception of unfairness, at work and beyond work, might have implications for functional decline in middle and older age. We recommend that – rather than addressing and changing individual perceptions of unfairness – more research is needed to find out whether specific environments can be defined as unfair and whether such environments can be effectively tackled in an attempt to truly improve public health.

## Background

In Denmark and Germany, the richest ten percent of people have a six times higher income than the poorest ten percent. In the United Kingdom and the United States, the richest have ten to fourteen times higher incomes than the poorest [1]. Income inequalities bring inequalities in housing conditions, neighbourhoods in which people live, circumstances at work, and number of material goods with which people can surround themselves [2]. These inequalities also bring inequalities in risks of disease and premature mortality [3]. Particularly because these socioeconomic inequalities have such profound implications for daily life, excesses in such distributive processes have raised heated scientific, ethical, and political discussions about whether the conditions in which people from lower socioeconomic groups live should be considered unfair [4,5].

Given this environmental background, it is striking to see that only so few examined whether a general perception of unfairness is particularly common in people who are allocated the smallest shares of resources [6]. Furthermore, only few have examined whether this perceived unfairness relates to subsequent poor health outcomes (whether it be through stress-related physiological mechanisms or through engaging in poor health behaviour) [6,7]. Most research in the field addressed injustice and perceived unfairness in the work situation only [8-11] or only used one item to measure perceived general unfairness [6,7]or did not examine perceived unfairness regarding its contribution to socioeconomic inequalities in health [7]. Using seven-year prospective cohort data from the Dutch SMILE study among 55 years olds and older, we set out to examine the contribution of perceived unfairness to the higher risks of physical and mental dysfunction in men and women with a lower socioeconomic position.

## Methods

### Study population

The Dutch SMILE study (i.e. Study on Medical Information and Lifestyles Eindhoven) is a prospective cohort study which started in November 2002 as a joint project of Maastricht University and the Eindhoven Corporation of Primary Health Care Centers [12]. Part of the data collection concerns people 55 years old and older. They received annual questionnaires in May. Medical diagnoses and deaths were registered via the computerised general practitioners’ registers. In May 2003, 9,557 persons of 55 years or older were sent a questionnaire, of whom 4,745 responded (49.7%). Of the respondents in 2003, 1,539 also responded on questionnaires in 2008 (when perceived unfairness was measured) and 2010 (when there was a repeat measurement of health-related functioning) (32.4%). After exclusion of the persons with missing scores on the relevant variables, the final sample comprised 1,282 participants (83.2%) (649 men (50.6%), 633 women (49.4%), mean age = 65; SD = 7). Participants were followed up for seven years. The study is approved by the medical ethical committee of the Maastricht Academic Hospital.

### Measures

#### Health-related functioning

Health-related functioning was measured by two summary scales of the SF-36; mental and physical functioning [13]. This SF-36 scale, which was included in the questionnaires of May 2003, 2008, and 2010, has thirty-six items on health, pain, health-related functioning, and psychosocial factors. The questions can be clustered into eight sub-scales which can be further recoded into two components, i.e. mental and physical functioning [14]. As per the SF-36 guideline, persons that had more than 50% missing scores in a subscale were considered missing; persons with less than 50% missing scores on a subscale were assigned the mean score of the other items. Functional change between 2003 and 2008 on the one hand and 2010 on the other hand was dichotomised. Functional decline was defined by having a change score that was more than 1 standard deviation below the average change (i.e. decline).

#### Socioeconomic status

In 2003, monthly household income was measured and subsequently individualised, taking into account the number of people living from that income [15]. The highest attained level of education was also asked for, looking at both the participant’s and his or her partner’s educational level. Participants were able to choose between eight different options from primary school to academic education. Income and education were correlated (Pearson correlation = 0.36; p-value: <0.001), but income had somewhat more missing values (3.4 versus 0.2 percent) and probably is less reliable than education. Both income and education were therefore standardised and the mean of both standardised scores was used to indicate socioeconomic status. This score was categorized into three categories based on tertiles: low, intermediate, and high level of socioeconomic status.

#### Perceived unfairness

The perception of unfairness was assessed in May 2008 by nine items. These items asked for how often people felt treated unfairly (Cronbach’s α = 0.85). In contrast to previous research [6-11]our questions refer to more general unfairness perceptions at work, but also beyond work. The nine items were: How many times you have/had the feeling that: a. you are being treated unfairly of unjustly?, b. important information is being withheld from you?, c. no-one is there to listen when you have problems?, d. no-one is there to offer actual help when you have problems?, e. you are being criticized wrongly?, f. you are not being valued for all your work?, g. other people are treated better or fairer than you?, h. the legislation in the Netherlands is disadvantaging you?, i. your efforts get too little reward? People could indicate whether they had this experience always (score 4), often (3), sometimes (2), rarely (1), or never (0). The mean across the nine items was computed. Missing scores on less than half of the scale were imputed with the mean of the other items for that person; cases with more than 50% missing scores were considered missing. Participants were subsequently classified in three categories bases on tertiles: low (mean score between 0 and 0.90), intermediate (0.90 and 1.34) and high perceived unfairness (1.34 and 4.00). The cut-offs indicate relatively low levels of perceived unfairness.

#### Covariates

Age and sex were measured in 2003 and were included in all the analyses as covariates.

### Statistical analysis

Depending upon whether variables were categorical or continuous, chi^2^-tests or F-tests were used to examine whether there were significant differences between socioeconomic groups in age, sex, perceived unfairness, mental and physical functioning in 2003, and their decline between 2003 and 2010. Multiple logistic regression models were estimated to examine how socioeconomic position and perceived unfairness scores were related to physical and mental decline (no, yes) between 2003 and 2010. These analyses were controlled for age, sex, and physical and mental functioning in 2003, respectively.

Several sensitivity analyses were performed to check the robustness of the findings. Firstly, sensitivity analyses were done to exclude a potential bias from the possibility that the responses to the perceived unfairness questionnaire in 2008 had been affected by prior functional decline (between 2003 and 2008). Therefore, the logistic regression analyses were controlled for health-related functioning in 2008; perceived unfairness was then related to functional decline between 2008 and 2010. Secondly, as the categorisation of socioeconomic position and perceived unfairness into three categories might have been too crude and the cut-offs too arbitrary [16]we re-ran all logistic regression analyses, but with the continuous forms of the socioeconomic and perceived unfairness variables. Thirdly, as also dichotomisation of the outcome variable (functional change) might have been too crude and too arbitrary [16]we also ran linear regression analyses (similar to the logistic regression analyses), but with the original continuous forms of longitudinal change in mental and physical function, socioeconomic position, and perceived unfairness.

## Results

Persons with a high socioeconomic position reported less perceived unfairness than persons with a low socioeconomic position (26 versus 39 percent) (Table 1). Furthermore, persons with a high socioeconomic position more often were men, more often were somewhat younger, had better scores on physical functioning at the start, and less often experienced a substantial decline in mental functioning than their counterparts with a lower socioeconomic position. There were no significant socioeconomic differences regarding baseline mental functioning (p = 0.10) and the decline in physical functioning (p = 0.35). 

**Table 1:** Baseline associations of socioeconomic position (SEP) with sex, age, perceived unfairness, and physical and mental decline between 2003 and 2010

	**Higher**	**Intermediate**	**Lower**	**P-value**^**a**^
	**SEP**	**SEP**	**SEP**	
	**(n = 443)**	**(n = 471)**	**(n = 368)**	
Men (%)	65.0	44.2	41.6	≤ 0.01
Age (mean)	64.9	64.4	65.9	0.01
High perceived unfairness (%)	25.5	33.1	38.9	≤ 0.01
2003 physical functioning (mean) ^b^	51.0	49.0	46.7	≤ 0.01
2003 mental functioning (mean) ^b^	50.4	50.4	49.4	0.10
Physical decline 2003–2010 (%)	16.0	13.4	12.8	0.35
Mental decline 2003–2010 (%)	9.3	10.0	16.3	≤ 0.01

^a^ P-values from chi^2^-test (categorical variables) and F-test (continuous variables).

^b^ Higher scores indicate better health-related functioning.

Multiplicative interactions of age and sex with socioeconomic position and perceived unfairness and of socioeconomic position with perceived unfairness did not show a consistent pattern of age or sex-specficity of our findings (not tabulated). Hence, no interactions were modeled in the subsequent analyses. Table 2 shows that perceptions of unfairness were related to both physical and mental decline between 2003 and 2010, although the latter only when perceived unfairness was introduced as a continuous score. Persons reporting much perceived unfairness had a 1.75 (95% confidence interval (CI): 1.16-2.62) and 1.48 (95% CI: 0.96-2.28) higher odds of physical and mental decline, respectively. Low socioeconomic position was related to mental decline, but not physical decline. Persons with a low socioeconomic position had a 1.82 higher odds of decline in mental functioning than persons with a high socioeconomic position (95% CI: 1.17-2.84). When both were controlled for each other, both odds ratios decreased, but not substantially. For example, above odds ratio of mental decline for low socioeconomic position decreased from 1.82 to 1.76 and remained statistically significant.

**Table 2:** Odds ratios (95% confidence intervals) of functional decline between 2003 and 2010 according to socioeconomic position (SEP) and perceived unfairness, adjusted for health-related functioning in 2003, age, and sex

	**Physical decline between ‘03 and ‘10**		**Mental decline between ‘03 and ‘10**	
	**Model 1**^**c**^	**Model 2**^**d**^	**Model 1**	**Model 2**
SEP				
High	1.00	1.00	1.00	1.00
Intermediate	0.90 (0.61, 1.32)	0.88 (0.60, 1.30)	1.05 (0.67, 1.66)	1.03 (0.65, 1.63)
Low	0.80 (0.53, 1.22)	0.76 (0.50, 1.16)	**1.82** (1.17, 2.84)	**1.76** (1.13, 2.75)
Continuous score ^a^	0.98 (0.80, 1.20)	0.94 (0.77, 1.15)	**1.33** (1.06, 1.67)	**1.30** (1.04, 1.64)
Perceived unfairness				
Low	1.00	1.00	1.00	1.00
Intermediate	1.32 (0.88, 1.99)	1.33 (0.88, 2.00)	1.09 (0.70, 1.69)	1.09 (0.70, 1.70)
High	**1.75** (1.16, 2.62)	**1.79** (1.19, 2.70)	1.48 (0.96, 2.28)	1.41 (0.91, 2.19)
Continuous score ^b^	**1.57** (1.17, 2.11)	**1.56** (1.16, 2.10)	**1.47** (1.07, 2.03)	**1.41** (1.02, 1.94)

^a^ The socioeconomic continuous score is a standardised score with mean 0 and standard deviation 1; higher scores indicate worse socioeconomic status. ^b^ The perceived unfairness continuous score ranges from 0 (low) to 4 (high). ^c^ Model 1: socioeconomic position and perceived unfairness not simultaneously adjusted. ^d^ Model 2: socioeconomic position and perceived unfairness simultaneously adjusted.

When only the change between 2008 and 2010 is considered (Table 3), a similar pattern of results is found, although perceived unfairness appears to lose its statistical significance when related to physical decline and when controlled for socioeconomic position. When all variables are introduced as continuous measures (Table 4), a similar pattern is found, although now the only non-significant finding is that of the association between low socioeconomic position and physical decline. Regression coefficients for both socioeconomic position and perceived unfairness decreased only little, when simultaneously controlled; both foremost have independent effects on health-related decline.

**Table 3:** Odds ratios (95% confidence intervals) of functional decline between 2008 and 2010 according to socioeconomic position (SEP) and perceived unfairness, adjusted for health-related functioning in 2008, age, and sex

	**Physical decline between ‘08 and ‘10**		**Mental decline between ‘08 and ‘10**	
	**Model 1**^**c**^	**Model 2**^**d**^	**Model 1**	**Model 2**
SEP				
High	1.00	1.00	1.00	1.00
Intermediate	1.18 (0.78, 1.79)	1.19 (0.78, 1.80)	1.45 (0.91, 2.31)	1.41 (0.88, 2.24)
Low	1.26 (0.82, 1.95)	1.24 (0.80, 1.93)	**1.99** (1.24, 3.17)	**1.88** (1.18, 3.02)
Continuous score ^a^	1.18 (0.95, 1.47)	1.16 (0.94, 1.45)	**1.30** (1.03, 1.64)	**1.27** (1.01, 1.59)
Perceived unfairness				
Low	1.00	1.00	1.00	1.10
Intermediate	1.38 (0.91, 2.10)	1.39 (0.91, 2.11)	1.10 (0.70, 1.75)	1.10 (0.69, 1.74)
High	1.33 (0.86, 2.04)	1.30 (0.84, 2.01)	**1.64** (1.05, 2.57)	1.54 (0.98, 2.42)
Continuous score ^b^	1.23 (0.90, 1.67)	1.19 (0.87, 1.62)	**1.46** (1.04, 2.04)	1.39 (0.99, 1.94)

^a^ The socioeconomic continuous score is a standardised score with mean 0 and standard deviation 1; higher scores indicate worse socioeconomic status. ^b^ Perceived unfairness continuous score ranges from 0 (low) to 4 (high). ^c^ Model 1:socioeconomic position and perceived unfairness not simultaneously adjusted. ^d^ Model 2: socioeconomic position and perceived unfairness simultaneously adjusted.

**Table 4:** Unstandardised regression coefficients (95% confidence intervals) of the association between functional change between 2003/2008 and 2010 and socioeconomic position and perceived unfairness, adjusted for health-related functioning in 2003/2008, age, and sex.^a^

	**Decline in physical functioning**		**Decline in mental functioning**	
	**Model 1**^**b**^	**Model 2**^**c**^	**Model 1**	**Model 2**
Decline between 2003 and 2010				
Socioeconomic position	0.14 (−0.67, 0.39)	0.05 (−0.58, 0.48)	**0.80** (0.32, 1.28)	**0.63** (0.15, 1.10)
Perceived unfairness	**1.20** (0.42, 1.97)	**1.19** (0.41, 1.97)	**2.01** (1.29, 2.72)	**1.88** (1.16, 2.59)
Decline between 2008 and 2010				
Socioeconomic position	0.29 (−0.17, 0.75)	0.18 (−0.28, 0.64)	**0.61** (0.54, 0.64)	**0.51** (0.06, 0.96)
Perceived unfairness	**1.21** (0.54, 1.88)	**1.18** (0.50, 1.85)	**1.26** (0.57, 1.94)	**1.16** (0.46, 1.85)

^a^ The socioeconomic continuous score is a standardized score with mean 0 and standard deviation 1; higher scores indicate worse socioeconomic status; the perceived unfairness continuous score ranges from 0 (low) to 4 (high); functional change is within-person change score in continuous form. ^b^ Model 1: socioeconomic position and perceived unfairness not simultaneously adjusted. ^c^ Model 2: socioeconomic position and perceived unfairness simultaneously adjusted.

## Discussion

Addressing general perceptions of unfairness both in work and beyond work, we found that middle-aged and older persons with a low socioeconomic position more often reported such perceptions than persons with a high socioeconomic position. Both socioeconomic position and perceived unfairness were related to health-related decline in mental functioning in the seven-year follow-up interval. Perceived unfairness was also related to health-related decline in physical functioning, but low socioeconomic position was not. When socioeconomic position and perceived unfairness were simultaneously controlled, odds ratios for both determinants decreased only very little. Hence, despite their association, perceived unfairness and low socioeconomic position foremost had independent associations with the outcomes.

Unfairness, at least the perception thereof, is an important predictor of functional decline. The question rises whether it is the subjective perception in itself that matters for health or whether there is something unfair about the environments in which these perceptions of unfairness are rooted. Despite perceived unfairness being more common in lower socioeconomic status groups (39 versus 26 percent), only a small part of the odds ratio for perceived unfairness was “explained” by our measure of socioeconomic position. This indicates the possibility of residual confounding by socioeconomic factors. Maybe more specific unfair situations would have provided more insight into the environmental roots of the perceptions of unfairness. For example, effort-reward imbalance at work and at home might prove one of the more specific routes through which unfair environments influence perceptions of unfairness and ultimately individual health-related functioning [17,18]. Similarly, unfairness perceptions might also be fuelled by the unfairness of restricted opportunities for upward social mobility [19]living in toxic areas [20]or being stigmatised and discriminated [21]. In short, more research is needed to find out whether in order to improve public health, it is more effective to have persons change their frustrating perceptions or to have the fundamental environmental unfairness and injustice diminished.

### Methodological issues

First, non-response at baseline is substantial, as is the attrition between 2003 and 2010. Non-respondents and those who left the cohort were more often of a low socioeconomic position, more often reported perceived unfairness, and more often reported poor functioning (not tabulated). This pattern might have contributed to an underestimation of the strength of the associations in the current report. Of the 1,282 participants, 36% had their SF-36 data imputed (less than 10% per sub-scale), as per the SF-36 guideline; most of whom with only one item missing. The simple personal mean imputation remains a viable and appropriate approach using the SF-36 [22]. Furthermore, some might have died prior to baseline and others have died during follow-up. Mortality, as an extreme form of functional decline, might also have resulted in underestimated associations in our report. However, both perceived unfairness and socioeconomic position were not related to the risk of mortality between 2008 and the end of 2010 (not tabulated).

Second, our study relied solely on self-reports, which may have led to information bias in our perceived unfairness and functioning measures. Individuals with a general tendency toward complaining may over-report perceived unfairness and mental or physical dysfunction. This may have led to an overestimation of the association between perceived unfairness and health-related functioning. However, we used longitudinal data and controlled for functioning at baseline in 2003 (and 2008). Next to excluding reversed causation, we assume that this will have also eliminated bias due to excessive complaining. More research is needed regarding how differential item functioning might additionally have affected our findings [23].

Third, in the absence of validated cut-offs, the cut-offs used in the logistic regression analyses might have been too crude and too arbitrary [16]. For the SF-36 outcome, we defined decline as reporting a decline that at least is one standard deviation more than the mean decline. Using two standard deviations would have left two few “decliners”. The findings of the sensitivity analyses using the original continuous variables confirm the general pattern of findings.

## Conclusion

Our findings suggest that the general perception of unfairness, at work and beyond work, might have substantial implications for both physical and mental functional decline in middle and older age. Although, in our cohort, perceived unfairness is more common in low socioeconomic positions, both have foremost independent effects on functional decline. However, rather than addressing and changing individual perceptions of unfairness, more research is needed to find out whether environments can be defined as unfair and whether such environments can be effectively tackled in an attempt to truly improve public health.

## Competing interests

The authors declare that they have no competing interests.

## Authors’ contributions

HB was the principal investigator, conducted the final data analyses, wrote the final drafts, and will act as guarantor for the paper. AG conducted the first analyses and wrote the first drafts. MvdA contributed to the management of the data collection. GK and HB developed the perceived unfairness questionnaire. All authors contributed to the interpretation of data and to critically reviewing all drafts. All authors read and approved the final manuscript.

## Pre-publication history

The pre-publication history for this paper can be accessed here:

http://www.biomedcentral.com/1471-2458/12/818/prepub
